# Antimicrobial resistance control in the emergency department: a need for concrete improvement

**DOI:** 10.1186/s13756-022-01135-6

**Published:** 2022-07-08

**Authors:** Martin Pin, Rajan Somasundaram, Christian Wrede, Frank Schwab, Petra Gastmeier, Sonja Hansen

**Affiliations:** 1 Florence-Nightingale-Hospital, Kaiserswerther Diakonie, Department of Emergency Medicine, Düsseldorf, Germany; 2 German Association for Emergency Medicine, (Deutsche Gesellschaft Interdisziplinäre Notfall- und Akutmedizin e.V., DGINA), Berlin, Germany; 3 Charité – Universitätsmedizin Berlin, Corporate Member of Freie Universität Berlin and Humboldt-Universität zu Berlin, Department of Emergency Medicine, Campus Benjamin Franklin, Berlin, Germany; 4 Helios Hospital Berlin-Buch, Department of Emergency Medicine, Berlin, Germany; 5 Charité – Universitätsmedizin Berlin, corporate member of Freie Universität Berlin, Humboldt-Universität zu Berlin, and Berlin Institute of Health, Institute of Hygiene and Environmental Medicine, Berlin, Germany; 6 National Reference Centre for Surveillance of Nosocomial Infections, Institute of Hygiene and Environmental Medicine, Berlin, Germany

**Keywords:** Antimicrobial resistance, Antibiotic use, Infection prevention and control, Emergency department

## Abstract

**Background:**

Rational use of antibiotics (AB) and infection prevention and control (IPC) are key measures for reducing antimicrobial resistance (AMR) in healthcare. Nonetheless, transferring evidence into clinical practice in emergency medicine has proven difficult. The extent to which structural requirements for implementing AMR control exist in German emergency departments (ED) was determined in a survey.

**Methods:**

Aspects of antimicrobial stewardship (AMS) and IPC implementation were surveyed within the German Association for Emergency Medicine (Deutsche Gesellschaft interdisziplinäre Notfall- und Akutmedizin e.V, DGINA) in 2018. Data were collected using an anonymous online questionnaire on ED characteristics, ED-based-link personnel for IPC and AMS, education and training, process monitoring and specific requirements for AMS and IPC as availability of AMR data and alcohol-based hand rub (AHR) consumption data. Data were analysed descriptively.

**Results:**

66 EDs with in median [interquartile range (IQR)] of 30,900 [23,000; 40,000] patient visits participated in the survey. EDs’ healthcare worker (HCW) received regular training on hand hygiene (HH) in 67% and on AMS in 20% of EDs. Surveillance of AHR consumption was performed by 73% EDs, surveillance of AB consumption by 64%. Regular audits on HH were performed in 39%. Training and audit activities, showed no significant variations according to EDs’ organizational characteristics. HCWs received immediate feedback of HH performance in 29%, in 23% a regular structured feedback of HH was provided. ED-based physicians with (1) specific IPC responsibilities and training were available in 61%, with (2) AMS training and responsibility in 15%. 83% had ED based IPC link nurses with precise ICP responsibilities in place. Essentially resistance data existed at the hospital level (74%) rather than at ED- or regional level (15% and 14% respectively).

**Conclusions:**

Management of AMR varies in German EDs, especially in accordance to hospital size and level of emergency care. IPC seems to receive more attention than AMS. Our data indicate the need for more implementation of regular IPC and AMS training in connection with monitoring and feedback in German EDs.

## Background

The global increase of antimicrobial resistance (AMR) reduces the effective treatment of infections and has a significant impact on patient morbidity and mortality [1, 2]. Addressing the rising threat of AMR various global and national efforts have been initialized [3, 4]. The two key measures in healthcare—the rational use of antibiotics and intense infection prevention and control (IPC) practices—have so far only been inadequately addressed in emergency care [5–9]. Particularly in emergency departments (ED), the strict attention of AMR is indicated in order to treat patients with an unknown multidrug- resistant organism (MDRO) carriage status adequately and to limit MDRO spread towards a growing number of vulnerable in- and outpatients receiving intensive diagnostics and therapy. The frequent need for in-time decision and administration of antibiotics in the ED requires the need for an effective empirical antibiotic therapy while avoiding unnecessary antibiotics and unnecessary selection pressure.

The fact that, depending on the specialty, up to 80% of inpatients are admitted via EDs points to the particular importance of EDs as a gateway for MDRO to hospitals and reinforces the need for concrete action already in this phase of medical treatment [10]. In addition, empirical therapy once started in the ED is often continued on the wards.

Various factors have been discussed as ED specific barriers to adoption of IPC measures [11, 12]. Barriers cited often by staff may more represent potential initial resistance to change rather than real barriers. Recent interventions showed that compliance improvement and even the invalidation of myths are possible in this clinical area [13–15]. Likewise some successful interventions have been described for AMS programmes in the ED [16, 17].

A continuous improvement of best practice realization apart from time-limited interventions requires a thorough structure for AMS and IPC. To what extent supporting structural requirements for AMR control exist in German EDs was investigated in a survey within the network of the German Association for Emergency Medicine (Deutsche Gesellschaft interdisziplinäre Notfall- und Akutmedizin e.V., DGINA). This article summarizes data on findings from 66 EDs.

## Methods

### Setting

Emergency departments in Germany were invited by email via the DGINA-network to take part in the survey from June to December 2018. Participation in the survey was based mainly on EDs’ interest rather than on a systematic sampling process.

### Data collection

A questionnaire with 11 items regarding IPC implementation and 9 items regarding AMS implementation was developed. Furthermore, data on MDRO screening and blood culture (BC) sampling, general characteristics such as hospital size, EDs’ allocation, ED physicians’ team structure and level of emergency care were collected [18].

All data were provided anonymously by ED directors or their deputies and collected via an online-survey (lime survey version 2.0).

Consent for participation was implied by completion of the survey. Since no patient data were collected and all ED characteristics were obtained anonymously, approval by the ethics committee was not required.

### Data analysis

Descriptive data analysis was performed after checking data plausibility. Continuous data are presented in median including interquartile range (IQR); categorical parameters are summarized by percentage. Results were summarized as totals and strata of the following four organizational parameters: *hospital size* (< 400 beds; ≥ 400 beds), *level of emergency care* (level 1,2,3) according to the German Federal Joint Committee (G-BA) categories with level 1 representing basic emergency care and level 2 and 3 representing extended and comprehensive emergency care, respectively [18]. *EDs’ attribution to hospital management* and *ED physicians’ team structure* (1) a core team consisting of more than 50% physicians primarily based in the ED (= “mainly core team”); (2) teams with more than 50% of medical specialists delegated only for a limited time to the ED (= “mainly physicians seconded to the ED”). Differences were tested by Chi-square or Wilcoxon ranksum test.

## Results

355 EDs were invited to participate in the survey, of which 66 (19%) from 13 out of 16 German federal states participated. With a median [interquartile range (IQR)] of 30,900 [23,000; 40,000] patient visits participating EDs represent around 2 million patients visits per year. The majority of EDs ranked among level of emergency care 2 and 3 according to G-BA categories (39% and 42%, respectively). EDs in hospitals with less than 400 beds were exclusively ranked as GBA category 1 and 2 (42% and 58%, respectively) whereas EDs in hospitals with more than 400 beds were most often ranked as GBA category 3 (60%) (Table 1).

**Table 1:** Level of care, allocation and physicians team structure of participating emergency departments (n = 66)

	Level of care according to the G-BA categories^a^ n (%)			Attribution of ED n (%)			ED physicians’ team structure n (%)		
Hospital size	1 n = 12	2 n = 26	3 n = 28	Attributed to CMO n = 52	Attributed to clinical department n = 12	No data n = 2	Mainly core team n = 24	Mainly physicians seconded to the ED n = 30	No data n = 12
< 400 Beds n = 19	8 (42)	11 (58)	0 (0)	17 (90)^b^	0 (0)^b^	2 (11)^b^	3 (16)	8 (42)	8 (42)
≥ 400 Beds n = 47	4 (9)^b^	15 (32)^b^	28 (60)^b^	35 (75)^b^	12 (26)^b^	0 (0)^b^	21 (45)^b^	22 (47)^b^	4 (9)^b^

G-BA, German Federal Joint Committee; ED, emergency department, CMO, Chief Medical Officer

^a^Level of emergency care according to the G-BA categories 1–3 [18]

^b^Not 100% in total due to rounding

Various ED physicians´ team structures were reported from 54 EDs (no data from 12 EDs; Table 1): (1) teams with more than half of physicians permanently based in the ED (“core team”, 24/54, 44%), (2) teams with more than half of medical specialists delegated only for a limited time to the ED either complemented (14/54, 26%) or not complemented (16/54, 30%) by a group (less than half) of physicians permanently based in the ED (latter both groups are summarized to one group (”mainly physicians seconded to the ED”, 30/54,  56%).

**Fig. 1:**
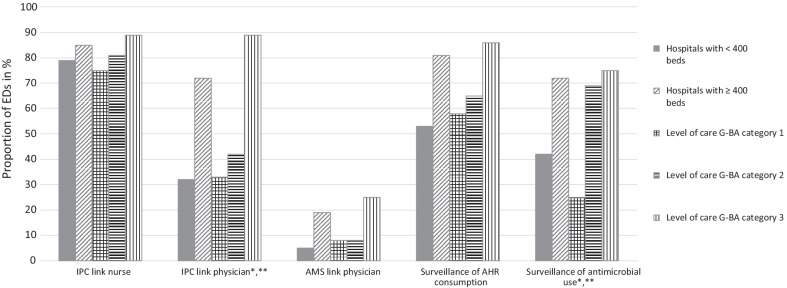
Proportion of emergency departments (EDs) with specifically trained and responsible ED-based link personnel and surveillance of process indicators for infection prevention and control (IPC) and rational antibiotic use (antimicrobial stewardship, AMS) in %, (n = 66). AHR: alcohol-based hand rub; G-BA: German Federal Joint Committee [18]. Hospital size (number of acute care beds); < 400 beds (n = 19), ≥ 400 beds (n = 47). Level of emergency care according to G-BA categories; Level 1 (n = 12), level 2 (n = 26), level 3 (n = 28). *Differences between size of the hospital: *P* < 0.05 (chi-square test). **Differences between level of emergency care according to the G-BA level categories: *P* < 0.05 (chi-square test)

Table 2 summarizes the IPC infrastructure. The majority of respondents reported that ED based link nurses with precise IPC responsibilities were available in 83%. Correspondent ED based IPC link physicians were reported less often by 61% of the EDs and their availability was indicated more often by EDs of larger hospitals (> 400 beds) and higher level of emergency care (*P* < 0.05) (Fig. 1).

**Table 2:** Infection control and prevention (IPC) characteristics of participating emergency departments stratified according to hospital size, level of care and organizational structure

	All (n = 66)	Hospital size^a^		Level of care according to the G-BA categories^b^			Organizational structure of ED^c^					
							Attribution of ED			ED physicians’ team structure		
		< 400 beds (n = 19)	≥ 400 beds (n = 47)	1 (n = 12)	2 (n = 26)	3 (n = 28)	Attributed to CMO (n = 51)	Attributed to clinical department (n = 11)	No data (n = 4)	Mainly core team (n = 23)	Mainly physicians seconded to the ED (n = 30)	No data (n = 13)
	n (%)											
IPC link physician^d,e^	40 (61)	6 (32)	34 (72)	4 (33)	11 (42)	25 (89)	31 (61)	7 (64)	2 (50)	14 (61)	19 (63)	7 (54)
IPC link nurse^f^	55 (83)	15 (79)	40 (85)	9 (75)	21 (81)	25 (89)	46 (90)	8 (73)	1 (25)	19 (83)	24 (80)	12 (92)
IPC Guidelines available for medical and nursing staff	66 (100)	19 (100)	47 (100)	12 (100)	26 (100)	28 (100)	51 (100)	11 (100)	4 (100)	23 (100)	30 (100)	13 (100)
Guidelines include WHO’s model "My 5 Moments for Hand Hygiene”	55 (83)	16 (84)	39 (83)	9 (75)	20 (77)	26 (93)	44 (86)	7 (64)	4 (100)	21 (91)	23 (77)	11 (85)
AHR dispenser availability immediately accessible at every treatment place / bed place	59 (89)	19 (100)	40 (85)	12 (100)	24 (92)	23 (82)	47 (92)	8 (73)	4 (100)	21 (91)	27 (90)	11 (85)
Use of AHR pocket or belt bottles												
For ≥ 50% of staff^g^	4 (6)	0 (0)	4 (9)	1 (8)	1 (4)	2 (7)	4 (8)	0 (0)	0 (0)	4 (17)	0 (0)	0 (0)
Rarely	28 (42)	8 (42)	20 (43)	7 (58)	10 (39)	11 (39)	21 (41)	5 (46)	2 (50)	11 (48)	13 (43)	4 (31)
Hand hygiene training offered in the ED												
Regularly at least once a year	44 (67)	13 (68)	31 (66)	6 (50)	18 (69)	20 (71)	35 (69)	7 (64)	2 (50)	17 (74)	21 (70)	6 (46)
Irregularly	16 (24)	5 (26)	11 (23)	4 (33)	5 (19)	7 (25)	13 (26)	2 (18)	1 (25)	4 (17)	5 (17)	7 (54)
Audits of hand hygiene carried out in the ED												
Regularly at least once a year	26 (39)	7 (37)	19 (40)	5 (42)	9 (35)	12 (43)	22 (43)	3 (27)	1 (25)	12 (52)	11 (37)	3 (23)
Irregularly	16 (24)	6 (32)	12 (26)	2 (17)	7 (27)	9 (32)	13 (26)	3 (27)	2 (50)	5 (22)	8 (27)	5 (39)
Feedback of hand hygiene audit observation data^h^												
Immediately during audit^g^	19 (29)	3 (16)	16 (34)	2 (17)	6 (23)	11 (39)	16 (31)	2 (18)	1 (25)	11 (48)	7 (23)	1 (8)
As part of regular structured feedback	15 (23)	4 (21)	11 (23)	3 (25)	5 (19)	7 (25)	12 (24)	2 (18)	1 (25)	6 (26)	7 (23)	2 (15)
As part of irregular structured feedback	11 (17)	4 (21)	7 (15)	3 (25)	2 (8)	6 (21)	7 (14)	3 (27)	1 (25)	4 (17)	5 (17)	2 (15)
Surveillance of AHR consumption in the ED	48 (73)	10 (53)	38 (81)	7 (58)	17 (65)	24 (86)	29 (57)	8 (73)	2 (50)	19 (83)	22 (73)	7 (54)
Feedback of AHR consumption data	33 (69)	8 (80)	25 (66)	5 (71)	10 (59)	18 (75)	26 (90)	7 (88)	0 (0)	14 (74)	15 (68)	4 (57)

G-BA, German Federal Joint Committee; ED, Emergency department; CMO, Chief Medical Officer; IPC, Infection control and prevention; WHO, World Health Organization; AHR, alcohol-based hand rub

^a^Hospital size (number of acute care beds); < 400 beds (n = 19), ≥ 400 beds (n = 47)

^b^Level of emergency care according to the G-BA categories; Level 1 (n = 12), level 2 (n = 26), level 3 (n = 28) [18]

^c^Organizational structure of ED: Attribution of ED; attributed to CMO (n = 51), attributed to clinical department (n = 11), no data (n = 4). Medical team structure; mainly core team (n = 23), mainly physicians seconded to the ED (n = 30), no data (n = 13)

^d^Differences between size of the hospital: *P* < 0.05 (chi-square test)

^e^Differences between level of emergency care according to the G-BA level categories: *P* < 0.05 (chi-square test)

^f^Differences between attribution of ED: *P* < 0.05 (chi-square test)

^g^Differences between medical team structures: *P* < 0.05 (chi-square test)

^h^Multiple answers possible

**Fig. 2:**
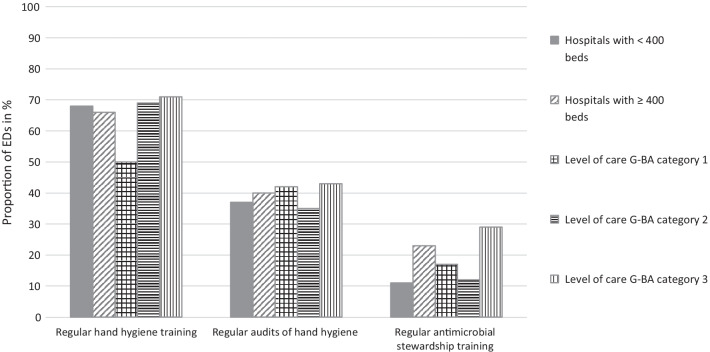
Proportion of emergency departments with regular training and auditing of hand hygiene and training of rational antibiotic use (antimicrobial stewardship) in %, (n = 66). Regular training and auditing was defined as “regular, at least once a year”. Hospital size (number of acute care beds); < 400 beds (n = 19), ≥ 400 beds (n = 47). Level of emergency care according to the German Federal Joint Committee (G-BA) categories; Level 1 (n = 12), level 2 (n = 26), level 3 (n = 28) [18]

The majority of EDs (89%) stated accessibility of AHR at every treatment place. The WHO model "My 5 Moments for Hand Hygiene” was part of local IPC guidelines in 83%, regular hand hygiene (HH) training at least once a year was offered in 67%; HH audits were carried out regularly at least once a year in 39% (Fig. 2).

Both, training and audit activities, showed no significant variations according to EDs’ level of care or general organizational structure. In 29% of EDs performing HH audits an immediate performance feedback was provided; more often in EDs with a physician “core team” structure (11/19) compared to those where more than 50% of ED physicians were delegated for a limited time to the ED (7/19; *P* < 0.05, Table 2). Regular structured feedback rounds of audit results were offered in 23%. Surveillance of AHR consumption was indicated by a large majority of EDs (73%), EDs’ healthcare worker (HCW) received feedback on the consumption data in a similarly high proportion (69%).

As shown in Table 3, the majority of EDs surveyed their AB use (64%); surveillance of AB use varied significantly by hospital size and level of emergency care (*P* < 0.05) (Fig. 1), but not by EDs’ organizational structure. Thirty percent of EDs mentioned no AB restriction policy; in case of restriction policies AB use was authorized by ED head physician or deputy (30%), followed by approval via specific prescription and inhibition of restricted antimicrobials at the hospital level (both 23%). Availability of resistance data varied significantly by level of emergency care (*P* < 0.05). Most often resistance data existed at the hospital level (74%) rather than at ED- or regional level (15% and 14%, respectively). An AMS expert was present at the hospital level in 74% and in 15% at the ED level. Regular AMS training was provided at least once a year by 20% of EDs, whereas the majority of EDs reported irregular training (35%; Fig. 1 and Table 3). Specific training on diagnostic stewardship was offered by 42% with significant variation depending on level of care and medical team structure (*P* < 0.05).

**Table 3:** Implementation of antimicrobial stewardship in participating emergency departments stratified according to hospital size, level of care and organizational structure

	All (n = 66)	Hospital size^a^		Level of care according to the G-BA categories^b^			Organizational structure of ED^c^					
							Attribution of ED			ED physicians’ team structure		
		< 400 Beds (n = 19)	≥ 400 beds (n = 47)	1 (n = 12)	2 (n = 26)	3 (n = 28)	Attributed to CMO (n = 51)	Attributed to clinical department (n = 11)	No data (n = 4)	Mainly core team (n = 23)	Mainly physicians seconded to the ED (n = 30)	No data (n = 13)
	n (%), if not otherwise specified											
AMS expert(s) at hospital level	49 (74)	14 (74)	35 (75)	7 (58)	21 (81)	21 (75)	41 (80)	6 (55)	2 (50)	16 (70)	22 (73)	11 (85)
Collaboration with ED to optimize antibiotic therapy	33 (67)	11 (79)	22 (63)	5 (71)	15 (71)	13 (62)	30 (73)	2 (33)	1 (50)	11 (69)	14 (64)	8 (73)
ED physician with AMS training and responsibility (AMS link physician)	10 (15)	1 (5)	9 (19)	1 (8)	2 (8)	7 (25)	9 (18)	1 (9)	0 (0)	5 (22)	4 (13)	1 (8)
Availability of resistance data^d^												
Resistance data at regional level	9 (14)	4 (21)	5 (11)	0 (0)	5 (19)	4 (14)	8 (16)	0 (0)	1 (25)	3 (13)	4 (13)	2 (15)
Resistance data at hospital level	49 (74)	14 (74)	35 (75)	6 (50)	21 (81)	22 (79)	41 (80)	6 (55)	2 (50)	15 (65)	23 (77)	11 (85)
Resistance data at ED level^e^	10 (15)	2 (11)	8 (17)	0 (0)	3 (12)	7 (25)	7 (14)	2 (18)	1 (25)	6 (26)	1 (3)	3 (23)
Surveillance of antimicrobial use^f,g^	42 (64)	8 (42)	34 (72)	3 (25)	18 (69)	21 (75)	34 (67)	7 (64)	1 (25)	15 (65)	19 (63)	8 (62)
Antimicrobial restriction policy in the ED^d^												
Authorisation by ED head physician or deputy	20 (30)	5 (26)	15 (32)	3 25)	7 (27)	10 (36)	18 (35)	1 (9)	1 (25)	9 (39)	8 (27)	3 (23)
Authorisation by local ED physician with AMS training and responsibility	1 (2)	0 (0)	1 (2)	0 (0)	1 (4)	0 (0)	1 (2)	0 (0)	0 (0)	1 (4)	0 (0)	0 (0)
Authorisation by hospital AMS expert(s)	4 (6)	2 (11)	2 (4)	0 0)	2 (8)	2 (7)	4 (8)	0 (0)	0 (0)	2 (9)	1 (3)	1 (8)
Approval via specific prescription^f^	15 (23)	2 (11)	13 (28)	0 (0)	5 (19)	10 (36)	12 (24)	2 (18)	1 (25)	7 (30)	6 (20)	2 (15)
Restricted antimicrobial is inhibited at the hospital level^e^	15 (23)	3 (16)	12 (26)	1 (8)	7 (27)	7 (25)	13 (26)	2 (18)	0 (0)	5 (22)	4 (13)	6 (46)
AMS training offered in the ED												
Regularly at least once a year	13 (20)	2 (11)	11 (23)	2 (17)	3 (12)	8 (29)	11 (22)	1 (9)	1 (25)	7 (30)	3 (10)	3 (23)
Irregularly	23 (35)	5 (26)	18 (38)	2 (17)	8 (31)	13 (46)	18 (35)	4 (36)	1 (25)	6 (26)	13 (43)	4 (31)
Diagnostic Stewardship training offered in the ED^e,f^	28 (42)	6 (32)	22 (47)	2 (17)	10 (39)	16 (57)	29 (57)	5 (46)	2 (50)	12 (52)	11 (37)	5 (39)
MDRO Screening in patients to be admitted to hospital^d^												
MRSA in general	12 (18)	2 (11)	10 (21)	1 (8)	6 (23)	5 (18)	9 (18)	3 (27)	0 (0)	2 (9)	7 (23)	3 (23)
MRSA according to risk factors^g^	45 (68)	17 (90	28 (60)	10 (83)	19 (73)	16 (57)	35 (69)	7 (64)	3 (75)	15 (65)	22 (73)	8 (62)
VRE in general	2 (3)	0 (0)	2 (4)	0 (0)	1 (4)	1 (4)	2 (4)	0 (0)	0 (0)	1 (4)	1 (3)	0 (0)
VRE according to risk factors	30 (46)	11 (58)	19 (40)	5 (42)	14 (54)	11 (39)	23 (45)	6 (55)	1 (25)	9 (39)	14 (47)	7 (54)
MRGN in general	3 (5)	0 (0)	3 (6)	0 (0)	1 (4)	2 (7)	2 (4)	1 (9)	0 (0)	1 (4)	2 (7)	0 (0)
MRGN according to risk factors	43 (65)	14 (74)	29 (62)	7 (58)	20 (77)	16 (57)	33 (65)	9 (82)	1 (25)	13 (57)	21 (70)	9 (69)
Numbers of blood cultures^h^ /100 patients; n of EDs providing data; median, (IQR)	29; 3.7 (2.4–7.9)	8; 4 (3.1–15.8)	21; 3.7 (1.9–7.3)	1; 1.2 (1.2–1.2)	15; 3.7 (3–8.1)	13; 6 (2.4–7.3)	23; 4.9 (2.8–8.1)	5; 3.1 (2.4–7.9)	1; 1.9 (1.9–1.9)	7; 2.8 (1.8–6.8)	16; 4.3 (2.7–8)	6; 8 (3.1–12)

G-BA, German Federal Joint Committee; ED, emergency department; CMO, Chief Medical Director; AMS, antimicrobial stewardship; MDRO, multidrug-resistant organisms; MRSA, methicillin-resistant *Staphylococcus aureus*; VRE, Vancomycin resistant Enterococci; MRGN, multidrug-resistant gram-negative bacteria

^a^Hospital size (number of acute care beds); < 400 beds (n = 19), ≥ 400 beds (n = 47)

^b^Level of emergency care according to the G-BA categories; Level 1 (n = 12), level 2 (n = 26), level 3 (n = 28) [18]

^c^Organizational structure of ED: Allocation of ED; Allocated to CMO (n = 51), allocated to clinical department (n = 11), no data (n = 4)

Medical team structure; mainly core team (n = 23), mainly physicians seconded to the ED (n = 30), no data (n = 13)

^d^Multiple answers possible

^e^Differences between medical team structures: *P* < 0.05 (chi-square test)

^f^Differences between level of emergency care according to the G-BA level categories: *P* < 0.05 (chi-square test)

^g^Differences between size of the hospital: *P* < 0.05 (chi-square test)

^h^Blood cultures were defined as a pair of one aerobic and one anaerobic bottle. In case of separate aerobe and anaerobe bottles, the number of aerobic bottles was selected

## Discussion

The study describes the results of a survey on selected structural requirements for IPC and AMS as the common approach for active AMR control. While HH as the universal IPC measure for healthcare-associated infections (HCAI) prevention has been known for decades, the evidence for rational antibiotic therapy has been shown more recently [19, 20]. Furthermore, HH is often primarily perceived as a self-protection measure [21, 22]. This could explain actual results of a high number of EDs providing IPC guidelines, AHR dispensers and ED based IPC link personnel and more HH training than AMS training. Despite the positive results with regard to equipment, it must be stated that effective AMR control relies on HCWs’ adherence with various measures and that guidelines and equipment alone do not necessarily lead to sufficient compliance with best practice [23].

Regular training and education are important in order to build up and continuously update HCWs’ knowledge on best practice. In addition, regular training demonstrates the need for individual competence and HCWs’ ownership for AMR control. Interestingly, with the exception of education for diagnostic stewardship, our data showed no significant differences in training activities in accordance to organizational characteristics as ED physician team structure or ED attribution. Unfavorable team structure is often mentioned as a barrier for implementation of best practice in the ED. Although recently “emergency medicine” (EM) has been implemented as supraspecialty, EM is not yet established as an own specialty in Germany and physicians staffing of German EDs is heterogeneous. Thus, continuous training is all the more important and ED leaders as well as IPC and AMS experts should rethink and intensify regular training offers despite potentially inhibiting organizational factors.

Generally, IPC education and training should be complemented by process verification through monitoring and auditing [24]. Regarding HH, German national guidelines recommend at least annual HH education and training and a regular evaluation and feedback of HCWs’ adherence [25]. An evaluation can take place in various ways. A simple way is the collection of AHR consumption data. Even if AHR consumption surveillance does not represent the gold standard in obtaining data on HCWs’ HH compliance it is a valuable surrogate parameter for HH performance and allows internal benchmarking over time and external benchmarking by taking national reference data into account [26]. Our data indicate, that many EDs collect data on AHR consumption. This was rather unexpected since beside and independent from our survey only about 60 German EDs took part in the corresponding national surveillance system’s module in the year of the survey—compared to 1.320 ICUs and 1.641 surgical non-ICU wards [27]. EDs which collect annual data of AHR consumption may consider to share these data and to contribute to more solid reference data by participating in the national surveillance system. National reference data on AHR consumption showed a median of 4 hand disinfection per patient (interquartile range 2; 6) [27]. More data on HH opportunities per patient in EDs are needed to interpret this average value for ED patients; the result of 4 hand disinfection per patient may be sufficient for patients with only very few HCW-patient contacts but not sufficient when considering a higher number of HH opportunities during intense patient care [28]. Since patient care provided in EDs shows huge varieties, data on AHR consumption should therefore be complemented by periodic auditing through direct compliance observations for an unequivocal data interpretation and to identify concrete gaps in HCWs’ adherence with HH [29]. In our survey, less than a half of EDs (39%) declared regular audits at least once a year.

An evaluation should be carried out also for AMS implementation. Auditing of physicians’ antibiotic prescribing practices may be an option to reduce the number of inappropriate prescriptions provided in the ED [30] but more commonly and easier to realize is a surveillance of AB consumption. Data on consumption are generally reported by pharmacists and help to identify concrete areas of improvement and to monitor and evaluate the impact of AMS interventions. Our data show that consumption data were more often collected in EDs in bigger hospitals and with a higher level of care. However, EDs should consider working with consumption data independently from size and level of care [31].

In addition to AB consumption data AMR data are important to support EDs’ AMS activities. The knowledge on AMR contributes to an effective empiric antibiotic therapy while taking local resistance into account. Concerning the availability of AMR data participating EDs most often stated the availability of resistance data at the hospital level and less often at the regional or ED level. Since hospital data may often include a broad spectrum of MDRO, hospital AMR data may not be applicable to most ED patients and may bear the risk of inadequate AB therapy. Thus, resistance data at the ED level are more helpful to estimate potential resistances in infected ED patients but are not always easily available. The combination of all data sets is certainly the best approach to consider antibiotic therapy for patients with community-acquired and with HCAI whereas usable regional data may replace ED-based data when these are difficult to obtain [32]. Future development and implementation of validated computer-based clinical decision support systems, connected to AMR data bases as well as to computerized physicians order entry may help emergency physicians, who work often under extreme time constraints, to decide on a right antibiotic therapy, reduce medication errors and thus, increase patients´ safety.

All monitored surveillance data on IPC and AMS are the basis for further action, e.g. education and training. Therefore, data should be used for immediate feedback at the individual level as well as for a time-delayed structured feedback at the team-level. As an example for increasing attention, Labricciosa et al. showed an increased awareness for AMR among emergency surgeons who were provided with periodic reports on local AMR data. In addition, HCWs who were receiving these reports considered poor HH more often an important cause of AMR [33]. As described by McClung et al. access to performance feedback under the umbrella of patient safety is a particularly motivating factor for HCW, far stronger than policies or regulatory considerations [34]. Interestingly, in our study HCWs in EDs with mainly medical core teams (> 50% of ED physicians based permanently in the ED) received more often immediate individual performance feedback during audits as HCWs in EDs with other medical team structures. Maybe these core teams were more open for feedback due to a grown team spirit and learning culture over time. During the last years, cultural aspects have been receiving an increasing importance in IPC and AMS implementation [24, 35]. Assessing department´s safety culture is very complex and thus, difficult to assess [36]. But although our survey did not directly address cultural aspects in the EDs, it might be hypothesized from our results that the relationship of medical team structure and reported performance feedback points to favorable management of safety culture in these EDs.

ED-based IPC link nurses and physicians with specific responsibilities and training can be supportive for implementing best practice by realizing and communicating department-specific characteristics to hospital-based AMS and IPC teams or committees and vice versa. Measures can be adapted and specific solutions can be introduced more sufficiently to local HCWs with the help of local link personnel. Ideally, link personnel are not merely appointed due to national recommendations [31, 37], but are intrinsically motivated and have some extra working time to facilitate local interventions as so called “champions” [38]. Our data show, that more EDs had IPC link nurses than IPC link physicians in place but since IPC is a topic for all professional groups and should be realized department-wide, adequate link personnel should be designated among nurses and physicians.

The survey is in line with other projects investigating the situation in EDs at a broader level [39–41]. Regarding these initiatives the necessary improvement of IPC and AMS deficits in emergency medicine may not exclusively be seen as an individual problem of individual EDs but as a general problem in healthcare which requires common solutions. EDs may be generally encouraged to prioritize AMR challenges and their efforts. Relevant data may be considered as standard quality indicators as already required in intensive care medicine [42]. To support EDs’ improvement locally these indicator and other process data should be relevant for hospitals’ infection control and or AMS committees and teams. To what extend the number of blood cultures represents a useful indicator for microbiological testing and diagnostic stewardship in EDs has to be further investigated.

As a major limitation of our study, participation was based on voluntary motivation rather than on representative sampling. Therefore, data are not fully representative for Germany and possibly reflect the situation in the EDs, which, through their involvement, deal particularly with the topics of IPC and AMS. Secondly, data were obtained by questionnaire and may overestimate the situation due to aspects of social desirability. Since data were surveyed anonymously data validation was not possible, but anonymity was considered with the purpose of a higher response rate among invited EDs. A strength of the survey is the interdisciplinary approach of ED and IPC experts offering a broad perspective on IPC and AMS necessity and feasibility in emergency medicine.

## Conclusions

To our knowledge, our survey offers a first overview of AMR management in German EDs. Despite some methodological limitations the findings demonstrate that there is a need for further structural improvement for AMR control in German EDs. Taking into account around 20 million ED visits in Germany our data should be a stimulus for further concrete measures, such as intensifying training and monitoring in cooperation with hospitals’ IPC and AMS teams. Furthermore, process evaluation data are needed and should be used for feedback to improve adherence with measures and thereby safety of care.

## Data Availability

The datasets used and/or analyzed during the current study are available from the corresponding author upon reasonable request.

## Abbreviations

AB: Antibiotics
AHR: Alcohol-based hand rub
AMR: Antimicrobial resistance
AMS: Antimicrobial stewardship
ARHAQ: Agency for Healthcare Research and Quality
BC: Blood culture
ED: Emergency department
DGINA: Deutsche Gesellschaft interdisziplinäre Notfall- und Akutmedizin e.V.
G-BA: Gemeinsamer Bundesausschuss
HCAI: Healthcare-associated infection
HCW: Healthcare worker
HH: Hand hygiene
IPC: Infection prevention and control
MDRO: Multidrug- resistant organism
WHO: World Health Organization

## References

[1] Cassini A, Diaz Högberg L, Plachouras D, Quattrocchi A, Hoxha A, Simonsen GS, Burden of AMR Collaborative Group (2019). Attributable deaths and disability-adjusted life-years caused by infections with antibiotic-resistant bacteria in the EU and the European Economic Area in 2015: a population-level modelling analysis. Lancet Infect Dis.

[2] Smith R, Coast J (2013). The true cost of antimicrobial resistance. BMJ.

[3] World Health Organization. Global action plan on antimicrobial resistance. Geneva: World Health Organization; 2015. https://www.who.int/publications/i/item/9789241509763 . Accessed 10 Dec 2021.

[4] European Commission. A European one health action plan against antimicrobial resistance (AMR). 2016. https://ec.europa.eu/health/amr/antimicrobial-resistance_en /. Accessed 10 Dec 2021.

[5] Liang SY, Riethman M, Fox J (2018). Infection prevention for the emergency department: out of reach or standard of care?. Emerg Med Clin N Am.

[6] Seo HJ, Sohng KY, Chang SO, Chaung SK, Won JS, Choi MJ (2019). Interventions to improve hand hygiene compliance in emergency departments: a systematic review. J Hosp Infect.

[7] Pallin DJ, Camargo CA, Schuur JD (2014). Skin infections and antibiotic stewardship: analysis of emergency departement prescribing practices, 2007–2010. West J Emerg Med.

[8] May L, Cosgrove S, L'Archeveque M, Talan DA, Payne P, Jordan J (2013). A call to action for antimicrobial stewardship in the emergency department: approaches and strategies. Ann Emerg Med.

[9] Mistry RD, Newland JG, Gerber JS, Hersh AL, May L, Perman SM (2017). Current state of antimicrobial stewardship in children’s hospital emergency departments. Infect Control Hosp Epidemiol.

[10] David S, Reuter S, Harris SR, Glasner C, Feltwell T, Argimon S (2019). Epidemic of carbapenem-resistant Klebsiella pneumoniae in Europe is driven by nosocomial spread. Nat Microbiol.

[11] Barratt R, Gilbert GL, Shaban RZ, Wyer M, Hor SY (2020). Enablers of, and barriers to, optimal glove and mask use for routine care in the emergency department: an ethnographic study of Australian clinicians. Australas Emerg Care.

[12] Carter EJ, Wyer P, Giglio J, Jia H, Nelson G, Kauari VE (2016). Environmental factors and their association with emergency department hand hygiene compliance: an observational study. BMJ Qual Saf.

[13] Scheithauer S, Kamerseder V, Petersen P, Brokmann JC, Lopez-Gonzalez LA, Mach C (2013). Improving hand hygiene compliance in the emergency department: getting to the point. BMC Infect Dis.

[14] Arntz PRH, Hopman J, Nillesen M, Yalcin E, Bleeker-Rovers CP, Voss A (2016). Effectiveness of a multimodal hand hygiene improvement strategy in the emergency department. Antimicrob Resist Infect Control.

[15] LeMaster CH, Hoffart N, Benzer T, Schuur JD (2014). Implementing the central venous catheter infection prevention bundle in the emergency department: experiences among early adopters. Ann Emerg Med.

[16] Savoldi A, Foschi F, Kreth F, Gladstone BP, Carrara E, Eisenbeis S (2020). Impact of implementing a non-restrictive antibiotic stewardship program in an emergency department: a four-year quasi-experimental prospective study. Sci Rep.

[17] May L, Quiros AM, Oever JT, Hoogerwerf J, Schoffelen T, Schouten J (2021). Antimicrobial stewardship in the emergency department: characteristics and evidence for effectiveness of interventions. CMI.

[18] Gemeinsamer Bundesausschuss. Regelungen des Gemeinsamen Bundesausschusses zu einem gestuften System von Notfallstrukturen in Krankenhäusern. 2018. https://www.g-ba.de/downloads/62-492-1598/Not-Kra-R_2018-04-19_iK2018-05-19.pdf. Accessed 10 Dec 2021.

[19] Semmelweis IP (1861). Die Aetiologie, der Begriff und die Prophylaxis des Kindbettfiebers.

[20] Baur D, Gladstone BP, Burkert F, Carrara E, Foschi F, Döbele S (2017). Effect of antibiotic stewardship on the incidence of infection and colonisation with antibiotic-resistant bacteria and Clostridium difficile infection: a systematic review and meta-analysis. Lancet Infect Dis.

[21] Erasmus V, Brouwer W, van Beeck EF, Oenema A, Daha TJ, Richardus JH (2009). A qualitative exploration of reasons for poor hand hygiene among hospital workers: lack of positive role models and of convincing evidence that hand hygiene prevents cross-infection. Infect Control Hosp Epidemiol.

[22] Borg MA, Benbachir M, Cookson BD, Redjeb SB, Elnasser Z, Rasslan O (2009). Self-protection as a driver for hand hygiene among healthcare workers. Infect Control Hosp Epidemiol.

[23] Plambech MZ, Lurie AI, Ipsen HL (2012). Initial, successful implementation of sepsis guidelines in an emergency department. Dan Med J.

[24] World Health Organization. Guidelines on core components of infection prevention and control programmes at the national and acute care facility level. Geneva: World Health Organization; 2016. https://www.who.int/teams/integrated-health-services/infection-prevention-control/core-components. Accessed 10 Dec 2021. 27977095

[25] Kommission für Krankenhaushygiene und Infektionsprävention (KRINKO) beim Robert Koch-Institut (RKI) (2016). Händehygiene in Einrichtungen des Gesundheitswesens. Bundesgesundheitsbl.

[26] Wetzker W, Walter J, Bunte-Schönberger K, Schwab F, Behnke M, Gastmeier P (2017). Hand rub consumption has almost doubled in 132 German hospitals over 9 years. Infect Control Hosp Epidemiol.

[27] Nationales Referenzzentrum für Surveillance von nosokomialen Infektionen. Referenzdaten Modul HAND-KISS_F. 2018. https://www.nrz-hygiene.de/fileadmin/nrz/module/hand/201801_201812_HAND_F_Ref.pdf. Accessed 10 Dec 2021.

[28] Haac B, Rock C, Harris AD, Pineles L, Stein D, Scalea T (2017). Hand hygiene compliance in the setting of trauma resuscitation. Injury.

[29] Magnus TP, Marra AR, Camargo TZ, Victor Eda S, da Costa LS, Cardoso VJ (2015). Measuring hand hygiene compliance rates in different special care settings: a comparative study of methodologies. Int J Infect Dis.

[30] Karras D (2006). Antibiotic misuse in the emergency department. Acad Emerg Med.

[31] Arbeitsgemeinschaft der Wissenschaftlichen Medizinischen Fachgesellschaften (AWMF) Strategien zur Sicherung rationaler Antibiotika-Anwendung im Krankenhaus. Registernummer 092-001. 2019. https://www.awmf.org/leitlinien/detail/ll/092-001.html. Accessed 10 Dec 2021.

[32] Robert Koch-Institut. Antibiotika Resistenz Surveillance (ARS). https://ars.rki.de/Content/Database/ResistanceOverview.aspx. Accessed 10 Dec 2021.

[33] Labricciosa FM, Sartelli M, Correia S, Abbo LM, Severo M, Ansloni L (2018). Emergency surgeons’ perceptions and attitudes towards antibiotic prescribing and resistance: a worldwide cross-sectional survey. World J Emerg Surg.

[34] McClung L, Obasi C, Knobloch MJ, Safdar N (2017). Health care worker perspectives of their motivation to reduce health care-associated infections. Am J Infect Control.

[35] Hulscher ME, Grol RP, van der Meer JW (2010). Antibiotic prescribing in hospitals: a social and behavioural scientific approach. Lancet Infect Dis.

[36] van Vegten A, Pfeiffer Y, Giuliani F, Manser T (2011). Patient safety culture in hospitals: experiences in planning, organising and conducting a survey among hospital staff. Z Evid Fortbild Qual Gesundhwes.

[37] Kommission für Krankenhaushygiene und Infektionsprävention (KRINKO) beim Robert Koch-Institut (RKI) (2009). Personelle und organisatorische Vorraussetzungen zur Prävention nosokomialer Infektionen. Bundesgesundheitsbl.

[38] Hendy J, Barlow J (2012). The role of the organizational champion in achieving health system change. Soc Sci Med.

[39] Yanagizawa-Drott L, Kurland L, Schuur JD (2015). Infection prevention practices in Swedish emergency departments: results from a cross-sectional survey. Eur J Emerg Med.

[40] Kudo D, Sasaki J, Ikeda H, Shiino Y, Shime N, Mochizuki T (2018). A Survey on infection control in emergency departments in Japan. Acute Med Surg.

[41] Poole NM, Shapiro DJ, Fleming-Dutra KE, Hicks LA, Hersh AL, Kronman MP (2019). Antibiotic prescribing for children in United States emergency departments: 2009–2014. Pediatrics.

[42] Kumpf O, Braun JP, Brinkmann A, Bause H, Bellgardt M, Bloos F (2017). Quality indicators in intensive care medicine for Germany—third edition 2017. Ger Med Sci.

