# Correction: Antimicrobial resistance control in the emergency department: a need for concrete improvement

**DOI:** 10.1186/s13756-022-01168-x

**Published:** 2022-10-31

**Authors:** Martin Pin, Rajan Somasundaram, Christian Wrede, Frank Schwab, Petra Gastmeier, Sonja Hansen

**Affiliations:** 1 Florence-Nightingale-Hospital, Kaiserswerther Diakonie, Department of Emergency Medicine, Düsseldorf, Germany; 2 Deutsche Gesellschaft Interdisziplinäre Notfall- und Akutmedizin e.V., DGINA), German Association for Emergency Medicine, Berlin, Germany; 3 Department of Emergency Medicine, Charité – Universitätsmedizin Berlin, Corporate Member of Freie Universität Berlin and Humboldt- Universität zu Berlin, Campus Benjamin Franklin, Berlin, Germany; 4 Department of Emergency Medicine, Helios Hospital Berlin-Buch, Berlin, Germany; 5 Berlin Institute of Health, Institute of Hygiene and Environmental Medicine, Charité – Universitätsmedizin Berlin, corporate member of Freie Universität Berlin, Humboldt- Universität zu Berlin, Berlin, Germany; 6 National Reference Centre for Surveillance of Nosocomial Infections, Institute of Hygiene and Environmental Medicine, Berlin, Germany

**Antimicrobial Resistance & Infection Control (2022) 11:94**.

10.1186/s13756-022-01135-6.

The original article [1] contained errors in the attribution of corresponding authorship and affiliation to co-authors, Petra Gastmeier and Sonja Hansen which have since been corrected.

